# Systemic Oxidative Stress and Conversion to Dementia of Elderly Patients with Mild Cognitive Impairment

**DOI:** 10.1155/2014/309507

**Published:** 2014-01-12

**Authors:** Carlo Cervellati, Arianna Romani, Davide Seripa, Eleonora Cremonini, Cristina Bosi, Stefania Magon, Carlo M. Bergamini, Giuseppe Valacchi, Alberto Pilotto, Giovanni Zuliani

**Affiliations:** ^1^Section of Medical Biochemistry, Molecular Biology and Genetics, Department of Biomedical and Specialist Surgical Sciences, University of Ferrara, Via Borsari 46, 44121 Ferrara, Italy; ^2^Gerontology and Geriatric Research Laboratory, IRCCS Casa Sollievo Della Sofferenza, San Giovanni Rotondo, Viale Cappuccini 1, 71013 Foggia, Italy; ^3^Section of Internal Medicine, Gerontology, and Clinical Nutrition, Department of Medical Science, University of Ferrara, Via Savonarola 9, 44100 Ferrara, Italy; ^4^Department of Life Sciences and Biotechnology, University of Ferrara, Via Borsari 46, 44121 Ferrara, Italy; ^5^Department of Food and Nutrition, Kyung Hee University, 1 Hoegi-dong, Dongdaemun-gu, Seoul 130-701, Republic of Korea; ^6^Geriatrics Unit, Azienda ULSS 16 Padova, S. Antonio Hospital, Via Facciolati 71, 35127 Padova, Italy

## Abstract

Mild cognitive impairment (MCI) is regarded as a prodromal phase of late onset Alzheimer's disease (LOAD). It has been proposed that oxidative stress (OxS) might be implicated in the pathogenesis of LOAD. The aim of this study was to investigate whether a redox imbalance measured as serum level of hydroperoxides (i.e., by-products of lipid peroxidation) and/or serum antioxidant capacity might be predictive of the clinical progression of MCI to LOAD. The levels of these two markers were measured in 111 patients with MCI (follow-up: 2.0 ± 0.6 years), 105 patients with LOAD, and 118 nondemented healthy controls. Multivariate analysis adjusted for potential confounding factors, including age, gender, smoking, and comorbidities, showed a significant increase (*P* < 0.05) in baseline levels of OxS in MCI and LOAD as compared to cognitive healthy controls. No differences in either of OxS markers were found by comparing MCI patients who converted (*n* = 29) or not converted (*n* = 82) to LOAD. Overall, these results suggest that systemic OxS might be a precocious feature of MCI and LOAD. However, the role of OxS as an early prognostic marker of progression to LOAD needs further investigations.

## 1. Introduction

Mild cognitive impairment (MCI) is regarded as an intermediate state between normal aging and dementia [1]. This preclinical condition is characterized by short-term or long-term memory impairment which, at variance of dementia, is not associated with significant daily functional disability [2]. Importantly, almost one half of these individuals evolves to late onset Alzheimer's disease (LOAD), accounting for about 60% of the total cases of dementia in USA and Western countries [3].

In the last decades, the attention of the researchers has been intensely focused on the molecular mechanisms underlying the etiopathogenesis of LOAD in older individuals. These efforts have produced multiple proofs in support of a key role of oxidative stress (OxS) in the onset and development of LOAD [4, 5].

In physiological conditions, there is a balance between oxidant molecules, among which reactive oxygen species (ROS) are the most studied, and antioxidants species [6, 7]. OxS occurs when this balance shifts towards reactive species generation leading to cellular/tissue oxidative damage [8]. Growing *in vitro* and animal evidence [9–11] suggest that OxS might be the “armed hand” of the amyloid-*β* (A*β*) peptides aggregates, which are the main constituent of senile plaques in the brain of LOAD patients. Indeed, it has been shown that these peptides form oligomers that could exert neurotoxicity effects by enhancing ROS level in the brain [12]. More specifically, from these experiments it emerged that A*β* oligomers can directly generate H_2_O_2_ (through a cupper-dependent superoxide dismutase-like activity [13]), activate NADPH-oxidase in astrocytes, and induce ROS production in mitochondria, by modulating the activity of enzymes like A*β*-binding alcohol dehydrogenase and *α*-ketoglutarate dehydrogenase [12, 14].

Further proofs, although still controversial, of the association between LOAD and OxS have been gathered by human studies [15–17]. Most of these studies showed that, as compared with elderly controls, patients affected by MCI or LOAD display increased oxidative damage, in particular to lipids (peroxidation), along with decreased antioxidants levels in peripheral fluids [15, 17]. However, due to the cross-sectional design, most of these studies were unable to establish any cause-effect relationships between OxS and cognitive impairment or dementia.

To the best of our knowledge, the available literature lacks of longitudinal studies, based on large population sample, investigating the temporal relationship between OxS and dementia. To address this crucial issue, we conducted a prospective study with the aim of investigating whether baseline serum level of hydroperoxides (i.e., by-products of lipid peroxidation) and/or serum antioxidant capacity might be predictive in the clinical progression from MCI to LOAD.

## 2. Materials and Methods

### 2.1. Study Design

The present study was conducted according to the Declaration of Helsinki (World Medical Association, http://www.wma.net/), the guidelines for Good Clinical Practice (European Medicines Agency, http://www.ema.europa.eu/), and the guidelines Strengthening the Reporting of Observational Studies in Epidemiology guidelines (http://www.strobe-statement.org/), and it was approved by the local Ethic Committee for human experimentation.

Written informed consent for research was obtained from each patient or from relatives or a legal guardian.

Personal data and medical history were collected by a structured interview from patients and caregivers. All patients underwent a general and neurological examination. For neuropsychological assessment, all patients were given a battery of tests as previously described [18]. Routine analyses were performed to exclude causes of secondary cognitive impairment, including serum B_12_ vitamin, serum folate, liver function tests including ammonia, kidney function tests, thyroid function tests, blood cell count, and arterial oxygen saturation. Subjects affected by severe congestive heart failure, severe liver or kidney disease, severe chronic obstructive pulmonary disease, and cancer were excluded. There were no evidences of acute illnesses at the time of clinical observation and blood sampling; no subject was taking NSAIDS, antibiotics, or steroids at the time of recruitment.

Criteria used for the diagnosis of diabetes, arterial hypertension, and cardiovascular diseases (CVD) were reported elsewhere [18]. Smokers were defined as patients with present or previous significant history of smoking (>180 packs/years).

#### 2.1.1. Diagnosis of MCI

From 1 January 2006 to 31 December 2012, one hundred eleven patients with diagnosis of MCI consecutive referring to the Day Hospital Services for Cognitive Decline (University of Ferrara, Italy) or to the Geriatric Unit of the IRCCS Casa Sollievo della Sofferenza (San Giovanni Rotondo, Italy) and followed for a mean period of 2 years (2.0 ± 0.6 years) were enrolled. Further 88 MCI patients were added to the MCI group (total number of MCI: 199) in the cross-sectional analysis.

MCI was defined as the presence of short/long-term memory impairment, with/without impairment in other single or multiple cognitive domains, in an individual who did not meet the standardized criteria for dementia [2]. We also required that the patient with MCI would be still independent in the activities of daily living (ADLs). Subjects with MCI due to known causes (e.g., severe depression, extensive white matter pathology, severe vitamin B_12_ deficiency) had been excluded. MCI patients were divided into 2 subgroups on the basis of clinical evolution at follow-up: (A) 82 patients whose cognitive performance remained stable or slightly improved (MCI/MCI); (B) 29 patients converted to LOAD during follow-up (MCI/LOAD). The diagnosis of LOAD during the follow-up was made according to the NINCDS-ADRDA criteria [19].

#### 2.1.2. Diagnosis of LOAD

Trained geriatricians in 105 patients made diagnosis of LOAD according to the NINCDS-ADRDA criteria. Only patients with “probable” Alzheimer's disease were selected for the inclusion in the study in order to increase specificity. The Global Deterioration Scale ranged from stage 4 to stage 6.

#### 2.1.3. Cognitive Healthy Controls

One hundred eighteen normal older individuals (controls) without any evidence of dementia and without any functional disability attributable to cognitive impairment were included in the study.

### 2.2. Assays of Biochemical Parameters

Venous blood was collected from subjects upon an overnight fast, between 8.30 and 9.30 A.M. Each blood sample was then stored for one hour at room temperature and centrifuged (3000 ×g for 10 minutes) to obtain serum which was then divided into aliquots and stored at –80° until analysis.

Hydroperoxides were assessed by colorimetric assay based on the reaction between these lipid peroxidation by-products and N,N-dimethyl-para-phenylenediamine [20, 21]. This method is based on the ability of transition metals to catalyze the formation of alkoxide and peroxide from hydroperoxides. These two radicals strongly interact with the chromogenic reagent, producing a radical cation that absorbs at 505 nm. Briefly, for each subject, 20 *μ*L of serum or standard (H_2_O_2_) were added to a solution containing 1960 *μ*L of acetate buffer (pH 4.8) and 20 *μ*L of chromogen (0.0028 M). The solution was incubated (37°C) and then read for optical density after 1 and 4 minutes. The concentration of hydroperoxides was obtained by the average ΔA_505_/min and expressed as Carratelli Units (CU), where 1 CU corresponds to 0.023 mM of H_2_O_2_ [20, 21].

The total amount of nonenzymatic serum antioxidants (such as uric acid, ascorbic acid, and *α*-tocopherol) was spectrophotometrically determined by Ferric Reduction Antioxidant Power (FRAP) assay [22] with modifications described in our previous study [15]. FRAP method measures the ability of water and fat-soluble antioxidants to reduce ferric-tripyridyltriazine (Fe^3+^-TPTZ) to the ferrous form (Fe^2+^) which absorbs at 593 nm. Briefly, acetate buffer (pH 3.6), TPTZ (10 mM), and FeCl_3_ (20 mM) were mixed in the ratio 10 : 1 : 1 to give the working solution. 30 *μ*L of serum or standard (FeSO_4_) were added to 970 *μ*L of this solution. The reaction mixture was then incubated at room temperature for 6 minutes and the adsorbance value was recorded at 595 nm. The results of this assay were expressed as FRAP units, where 1 FRAP corresponds to 100 *μ*moles/L of Fe^3+^ reduced to Fe^2+^in 6 minutes.

FRAP assay is strongly influenced by the amount of uric acid, of which role as physiological antioxidant is highly controversial [23]. To overcome this bias, urate concentration was separately assessed by direct enzymatic method (in which urate is oxidized by uricase coupled with peroxidase [23]) and then subtracted from FRAP values. The resulting parameter (expressed in FRAP units), that is, residual antioxidant power (RAP), affords a reliable index of antioxidant status in uric acid-rich fluids such as serum [22, 24].

### 2.3. Brain Computer Tomography Scan

All patients (LOAD and MCI) underwent a brain Computer Tomography (CT). The instrument used was a third-generation SIEMENS SOMATON HQ. The slice thickness was 10 mm. Radiograms were evaluated by trained radiologists who were not informed about the clinical characteristics of the patient. The CT scan information was used to support the clinical diagnosis and to diagnose possible brain pathologies associated with secondary cognitive impairment.

### 2.4. Statistical Analysis

Means were compared by ANOVA and ANCOVA (Fisher's least significant difference as *post hoc* test), while prevalences were compared by the *χ* ^2^ test. Since the distribution of RAP was skewed, the values were log-transformed in order to approximate a normal distribution before entering univariate and multivariate analysis. The covariates included in the ANCOVA analysis for OxS markers were the following: age (years), gender (M/F), CVD (yes/no), diabetes (yes/no), hypertension (yes/no), and smoking habit (current, never). A two-tailed probability value <0.05 was considered statistically significant. SPSS 17.00 for Windows (Chicago, IL, USA) was used for statistical analysis.

## 3. Results

The main characteristics of the four groups of subjects enrolled into the study are reported in Table 1. MCI/MCI, MCI/LOAD, and LOAD patients were older and had a lower formal education level compared to controls. Both MCI groups presented a lower percentage of women compared with the other two groups, while the frequency of smokers did not consistently vary among groups. As expected by selection criteria, the average Mini Mental State Examination (MMSE) score was pathological in LOAD, while it was within normal limits in both MCI groups and controls. As regards comorbidities, CVD and diabetes were more frequent in the MCI groups compared with controls and LOAD, while hypertension was more frequent in LOAD patients with respect to controls.

**Figure 1:**
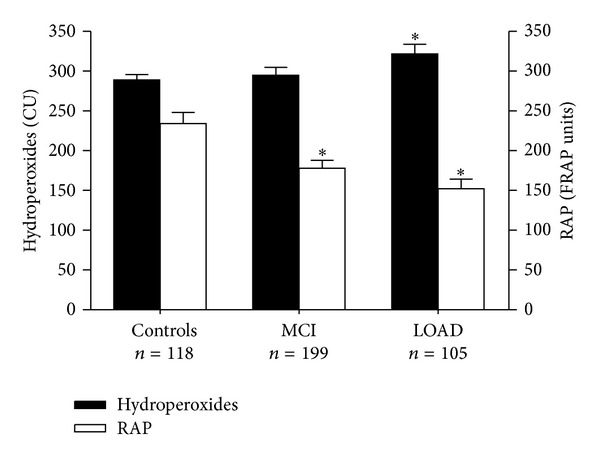
Mean levels of hydroperoxides and RAP in nondemented healthy controls, total MCI (MCI/MCI + MCI/LOAD + MCI no follow-up), and LOAD patients. CU = Carratelli Units; FRAP = Ferric reduction antioxidant capacity. In the ANCOVA model: age, gender, hypertension, cardiovascular diseases, diabetes, and smoking habit. *P < 0.01 versus controls.

In Table 2 are described the mean levels of serum hydroperoxides and residual antioxidant power (RAP) in controls, MCI/MCI, MCI/LOAD, and LOAD patients. Compared to healthy individuals, serum hydroperoxides were higher while RAP was lower in LOAD, after taking into account the possible effect of age, gender, smoking, and comorbidities (ANCOVA *post hoc* *P* < 0.01). Serum hydroperoxides were higher in MCI/MCI subjects than in controls (ANCOVA *post hoc* *P* < 0.05), while no difference in either OxS markers emerged by comparing the two MCI subgroups. When the whole group of MCI (111 with follow-up plus 88 without follow-up) was considered, a significant increase in hydroperoxides (ANCOVA *post hoc* *P* < 0.01) together with a significant reduction of RAP (ANCOVA *post hoc* *P* < 0.01) was observed compared with controls (Figure 1).

**Figure 2:**
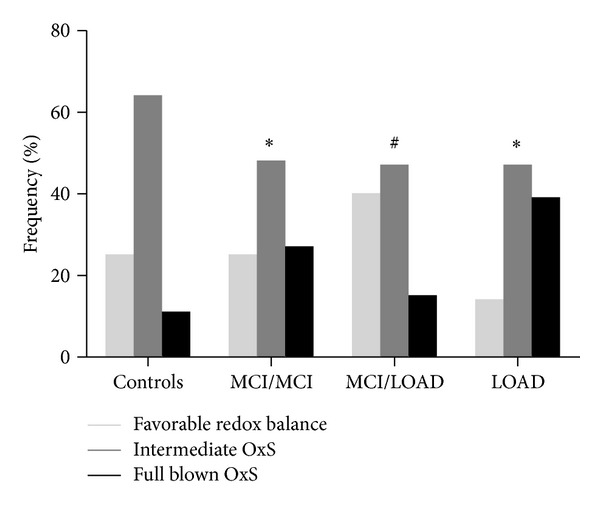
Within group percentages of subjects with Favorable Oxidative Balance, Intermediate OxS, or Full Blown OxS (for definitions see text). CU = Carratelli Units; FRAP = Ferric reduction antioxidant capacity. MCI/MCI: stable MCI patients. MCI/dementia: MCI patients converted to LOAD. *P < 0.01 versus Controls; ^#^ *P* < 0.01 versus LOAD.

Based on the median values of OxS markers from the whole sample (i.e., 305.0 CU for hydroperoxides, and 208.8 FRAP units for RAP) three subgroups of individuals were identified: (1) favourable redox balance: low hydroperoxides and high RAP; (2) intermediate OxS: high hydroperoxides and high RAP or low hydroperoxides and low RAP; (3) full blown OxS: high hydroperoxides and low RAP. In Figure 2 are reported the within sample group (Controls, MCI/MCI, MCI/LOAD, and LOAD) percentages of subjects with different degree of OxS. In line with the ANCOVA results, controls and LOAD displayed opposite proportions as regards the two extreme states of oxidative balance. Notably, the relative percentages of favourable redox balance and full blown OxS in MCI/dementia were markedly different (about three times for both) from those of LOAD patients.

## 4. Discussion

Most of the proofs supporting the involvement of OxS in LOAD development have been generated by experiments on cell cultures, animals and *postmortem* human brain tissues [9, 10, 25–27]. On the contrary, data from human studies are conflicting [15–17, 28, 29] and do not allow to draw a definitive picture about the role of OxS in the onset and progression of this neurodegenerative disorder.

In the present study, we evaluated the serum levels of hydroperoxides and RAP in a large sample of individuals including healthy controls, LOAD, and MCI patients that during 2-year follow-up remained either stable or converted to dementia. The main finding was that neither baseline level of the two peripheral markers was able to predict the progression from MCI to LOAD. In other words, the evaluation of systemic OxS by using these markers might not be a helpful tool in differencing MCI patients who are going to evolve to LOAD.

Overall, our data are consistent with those shown by one of the few other longitudinal studies employing a broad spectrum of nonenzymatic and enzymatic antioxidants, as well as lipid and protein oxidation markers [30]. In their prospective study conducted in a sample of 70 MCI subjects, Baldeiras et al. did not find significant differences in any of the baseline indexes of oxidative damage and antioxidant defence measured between stable and progressing to dementia individuals [30]. In contrast, in another study conducted on a similar number of subjects, F2-isoprostanes measured in cerebrospinal fluid (CSF) were significantly higher in MCI patients who later converted to LOAD compared to stable patients [31]. The discrepancies between these data and ours might be mainly due to the differences regarding the markers (and biological fluids) that were employed for OxS detection as well as regarding the general characteristics (e.g., age and lifestyle habits) of the population samples.

However, some important indications arise from the analysis of our cross-sectional data, which appear to confirm the results of previous works conducted on smaller samples [13, 17]. Our data clearly suggest from one side that OxS might represent an early event in LOAD pathogenesis and also that the process of redox balance derangement might take place in its prodromal phase (Figure 1). Consistently, mitochondrial dysfunction [32] and iron homeostasis dysregulation [27], which are believed to be the major causes of cumulative oxidative damage observed in neurons of LOAD [14], are also present in subject with MCI. Moreover, *postmortem* studies on brain tissues identified similar level of ROS by-products in proteins, lipids, and DNA from hippocampus and prefrontal regions in MCI and LOAD patients [33]. In particular, the identification of carbonylated and nitrated proteins common for MCI and Alzheimer's disease by redox proteomic approach suggests that key oxidative pathways can be an early event and playing, therefore, a role in the initial progression of the neurodegenerative process [34]. Oxidative protein damage is not random, but highly selective, and affects enzymes involved in energy metabolism (enolase, lactate dehydrogenase, creatine kinase, etc.), protein turnover (ubiquitin carboxyl terminal hydroxylase L1, UCHL-1), and control of excitotoxicity (glutamine synthetase) [35, 36].

Notably, the biomolecules present in neurons of these patients are highly vulnerable to oxidative challenge, because the low expression of endogen antioxidants (e.g., glutathione and coenzyme Q10) [34, 35]. This phenomenon is mostly due to a process (most probably caused by A*β* deposition) that leads to low expression of nuclear factor, E2-related factor 2 (Nrf2), that is responsible for activating transcription of antioxidant genes in the response to OxS [34, 36, 37].

The evidence of a similar oxidative and inflammatory [38] pattern in MCI and LOAD strengthen the widely supported concept of a biochemical equivalence between pre- and clinical conditions [39] which, in turn, might explain the lack of differences between MCI/MCI and MCI/dementia shown in our study.

In our opinion, our data adds to clinical practise, especially as it regards the use of antioxidant supplementation in the treatment of MCI and LOAD patients. Indeed, if confirmed on larger samples, the finding that OxS level might not be crucial for the progression from MCI to LOAD might explain the ineffectiveness of antioxidant therapy in MCI patients [40]. On the contrary, this approach would be possibly beneficial in healthy subjects that still do not present the signs of an altered oxidative balance.

Finally, we would like also to underline some limitations and strengths of this study. First, the full assessment of nutritional status was not performed in the subjects of this study. For this reason, it is difficult to establish, with a high degree of certainty, whether the decrease of RAP observed in MCI and LOAD patients is linked to a ROS-dependent depletion or to a scarce dietary intake of antioxidants. Second, we are aware that other markers of OxS might be more helpful than those we used in this study, for the understanding of the mechanism underlying the relationship between OxS and LOAD. In particular, the measurement of 4-hydroxynonenal (4HNE)-protein adducts could be used to address this aim, because of their proved ability to modulate signaling pathway [41]. Third, to the best of our knowledge, the sample MCI subjects enrolled for longitudinal study (*n* = 111), as well the size of the whole cross-sectional sample (*n* = 422), are by far the largest among human studies on this specific topic.

## 5. Conclusion

Taken together our data suggest that OxS might be precociously involved in LOAD pathogenesis. However, further investigations are needed to determine the appropriate OxS indicators to be measured in the progression from MCI to LOAD needs.

## Figures and Tables

**Table 1:** Principal characteristics of nondemented healthy controls, MCI/MCI, MCI/LOAD, and LOAD patients.

	Controls (*n* = 118)	MCI/MCI (*n* = 82)	MCI/LOAD (*n* = 29)	LOAD (*n* = 105)
Age (years)	69.5 ± 9.1	75.9 ± 6.7^c^	78.6 ± 5.7^c^	78.1 ± 5.5^c^
Female gender (%)	72.0^a,b^	54.8	51.7	70.1^a,b^
Formal education (years)	9.1 ± 4.3	5.9 ± 3.4^c^	6.4 ± 4.1^c^	5.3 ± 3.5^c^
MMSE score (/30)	26.7 ± 2.7	25.8 ± 2.9^b,c^	24.1 ± 2.3^a,c^	20.4 ± 4.4^a,b,c^
GDS (/15)	6.2 ± 3.5	5.9 ± 2.6	5.4 ± 3.4	5.4 ± 3.3
Hypertension (%)	42.1	55.1	54	64.1^c^
Diabetes (%)	10.1^a,b^	20.1	24.2	13.7^a,b^
CVD (%)	9.5^a,b^	25.6	25.1	16.7^a,b^
Smoking (%)	8.5	5.1	4.7	8.1

Continuous variables are expressed as mean ± standard deviation. MCI/MCI: stable MCI patients; MCI/LOAD: MCI patients converted to LOAD. CVD: cardiovascular disease; MMSE: Mini Mental State Examination; GDS: Global Deterioration Scale.

^a^ *P* < 0.05 versus MCI/MCI; ^b^ *P* < 0.05 versus MCI/LOAD; ^c^ *P* < 0.05 versus controls.

**Table 2:** Mean levels (mean ± standard error of the mean, SEM) of serum hydroperoxides and residual antioxidant power (RAP) in nondemented healthy controls, MCI/MCI, MCI/LOAD, and LOAD patients.

	Controls (*n* = 118)	MCI/MCI (*n* = 82)	MCI/LOAD (*n* = 29)	LOAD (*n* = 105)	ANCOVA (*P*)
Hydroperoxides (CU)	288.1 ± 11.9	295.8 ± 10.0^c^	281.2 ± 17.5	320.9 ± 12.9^c^	0.01
RAP (FRAP units)	234.5 ± 16.0	187.5 ± 16.2	205.1 ± 22.1	152.2 ± 15.5^c^	0.06

MCI/MCI: stable MCI patients; MCI/LOAD: MCI patients converted to LOAD. CU: Carratelli Units; FRAP: Ferric reduction antioxidant capacity. In the ANCOVA model: age, gender, hypertension, cardiovascular diseases, diabetes, and smoking habit.

^c^ *P* < 0.05 or *P* < 0.01 versus controls.
